# Characterization and Protective Properties of Lactic Acid Bacteria Intended to Be Used in Probiotic Preparation for Honeybees (*Apis mellifera* L.)—An In Vitro Study

**DOI:** 10.3390/ani13061059

**Published:** 2023-03-15

**Authors:** Aleksandra Leska, Adriana Nowak, Justyna Rosicka-Kaczmarek, Małgorzata Ryngajłło, Karolina Henryka Czarnecka-Chrebelska

**Affiliations:** 1 Department of Environmental Biotechnology, Faculty of Biotechnology and Food Sciences, Lodz University of Technology, Wolczanska 171/173, 90-530 Lodz, Poland; 2 Institute of Food Technology and Analysis, Faculty of Biotechnology and Food Sciences, Lodz University of Technology, Stefanowskiego 2/22, 90-537 Lodz, Poland; 3 Institute of Molecular and Industrial Biotechnology, Faculty of Biotechnology and Food Sciences, Lodz University of Technology, Stefanowskiego 2/22, 90-573 Lodz, Poland; 4 Department of Biomedicine and Genetics, Chair of Biology and Medical Microbiology, Medical University of Lodz, 5 Mazowiecka Str. (A-6 Building), 92-215 Lodz, Poland

**Keywords:** probiotics, *Apis mellifera* L., honeybee, SCFA, sugar syrup, antibiotics, survival, bile salts, cytotoxicity, mucin degradation

## Abstract

**Simple Summary:**

Honeybees (*Apis mellifera* L.) are economically and ecologically significant pollinators on a global scale. Various factors such as pathogens, insecticides, viruses, and mites increase colony mortality. The significance of honeybees makes it important to find ecological ways to strengthen the resistance of these insects. Lactic acid bacteria (LAB) are probiotic microorganisms that have a positive effect on the health of the host. Due to numerous beneficial properties, LAB have the potential to improve the viability of honeybees and conditions in apiaries. In this study, we examined the various properties of probiotic candidates such as survival in gastrointestinal conditions and sugar syrups, antibiotics resistance, organic acid profile, cytotoxicity of LAB metabolites, bile salts hydrolase activity, hydrogen peroxide production, β-hemolytic activity, insecticide detoxification by cell-free supernatants, mucin degradation ability and mutual antagonism between isolates. Most of the properties demonstrated by the isolates depended on the tested strain. The obtained results contributed to the selection of LAB to protect honeybees against various health risks.

**Abstract:**

Lactic acid bacteria (LAB) are widely used probiotics and offer promising prospects for increasing the viability of honeybees. Thus, the probiotic potential of 10 LAB strains was determined, which in our previous studies showed the most potent protective abilities. In the current study, we investigated various properties of probiotic candidates. The tested LAB strains varied in susceptibility to tested antibiotics. Isolates showed high viability in sugar syrups and gastrointestinal conditions. None of the LAB strains exhibited β-hemolytic activity, mutual antagonism, mucin degradation, hydrogen peroxide production capacity, or bile salt hydrolase (BSH) activity. Additionally, the cytotoxicity of LAB cell-free supernatants (CFS) was assessed, as well as the effect of CFS from *P. pentosaceus* 14/1 on the cytotoxicity of coumaphos and chlorpyrifos in the Caco-2 cell line. The viability of Caco-2 cells reached up to 89.81% in the presence of the highest concentration of CFS. Furthermore, LAB metabolites decreased the cytotoxicity of insecticides (up to 19.32%) thus demonstrating cytoprotective activity. All tested LAB strains produced lactic, acetic, and malonic acids. This research allowed the selection of the most effective LAB strains, in terms of probiosis, for future in vivo studies aimed at developing an ecologically protective biopreparation for honeybees.

## 1. Introduction

Western honeybee (*Apis mellifera* L.) is exposed to many factors contributing to increased colony mortality [1]. Factors affecting the viability of these pollinators include pesticides (e.g., coumaphos, chlorpyrifos, and neonicotinoids), pathogens (e.g., *Paenibacillus larvae*, *Nosema* spp., *Melissococcus plutonius*, and *Aspergillus* spp.), varroa mites, viruses, and environmental pollutants [1]. It is estimated that 87.5% of flowering plants are pollinated by animals [2]. *Apis mellifera* L. is the most frequent flower-visiting insect in the natural environment globally and some plant species (e.g., *Ornithogalum virens*, *Euryops laxus*, *Indigofera foliosa*, *Cyanotis speciosa,* and *Rubus ludwigii*) are exclusively pollinated by honeybees [3]. In order to improve the viability of honeybee colonies and prevent infections caused by pathogens, antibiotics are used in modern beekeeping to inhibit bacterial growth [4]. An example is tylosin used to treat *Paenibacillus larvae*, the causative agent of American foulbrood [4]. However, the use of antibiotics can have a negative impact on honeybee health and induce the mortality of these pollinators [5]. Dysbiosis caused by exposure to antibiotics can be reduced by administering probiotics [6]. According to an expert panel convened in 2013 by International Scientific Association for Probiotics and Prebiotics (ISAPP), probiotics can be defined as “live microorganisms which, when administered in adequate amounts, confer a health benefit on the host” [7]. Probiotic microorganisms strengthen the immune system, prevent infections, improve oral health, and regulate intestinal microbial balance [8]. Commonly used probiotics are strains of lactic acid bacteria (LAB) [9]. These gram-positive bacteria have a high tolerance to low pH and occur in the form of cocci or rods [10]. The metabolites produced by LAB inhibit the growth of pathogens, modulate the microbiota and strengthen the host’s immune system [11]. Products of LAB metabolism that perform antimicrobial functions include organic acids (e.g., lactic, fumaric, citric, and malic), bacteriocins, ethanol, CO_2_, diacetyl, H_2_O_2_, acetaldehyde, acetoin, and ammonia [12]. Lactic acid inhibits the growth of pathogens by disrupting the function of cell membranes, leading to lysis [13]. Some bacteriocins produced by *Lacticaseibacillus rhamnosus* contribute to a change in the pH gradient of the transmembrane and an increase in the permeability of the cell membrane, thereby strengthening host resistance to infections caused by pathogenic microorganisms [14]. However, the exact mechanism of action of most bacteriocins has not yet been elucidated [14]. The sensitivity of pathogens to LAB metabolites depends on the microorganism and environmental conditions (e.g., pH) [15].

LAB naturally colonize the gastrointestinal tract (GIT) of honeybees, inhibit the growth of various pathogens and contribute to the detoxification of chemical substances [16]. These non-pathogenic bacteria also stimulate egg-laying by the queen and increase the number of individuals in colonies [17]. The microbiota of honeybees plays a key role in the development of insects by supporting their vigor and health [18]. Using LAB as probiotics in the diet of these pollinators has many health benefits [17]. LAB increase the resistance of larvae and other life stages of honeybees, reducing the sensitivity to external factors [19]. Supplementation of honeybees with *Lactobacillus* strains provides a higher honey yield, contributing to economic improvement in beekeeping [20]. According to Cingeľová Maruščáková et al., *Levilactobacillus brevis* B50 increases the expression of pattern recognition receptors and genes encoding antimicrobial peptides (defensin-1 and abaecin) [21]. Probiotic LAB decrease colony mortality and cause positive physiological changes such as increased hypopharyngeal gland size in young honeybees [22]. Some LAB strains also alleviate dysbiosis caused by antibiotic treatment [23].

Due to the economic and environmental importance of pollinators, there is a growing need to find a way to improve the conditions in apiaries and increase the viability and resilience of honeybee colonies with natural, ecological methods. The topic of existing preparations for honeybees has been covered in our previously published review article [24]. LAB perform numerous beneficial functions in the honeybee body and are Generally Recognized as Safe (GRAS) microorganisms [25]. Thus, this research involves testing LAB strains isolated mostly from honeybee environments which in our previous studies showed the most potent abilities such as the utilization of carbohydrates, adhesion to biotic and abiotic surfaces, auto-aggregation, hydrophobicity, detoxification of pesticides, and antagonistic activity against honeybee pathogens [26,27,28,29].

The purpose of the current study was to investigate various properties of probiotic candidates for honeybees such as antibiotic resistance, survival in gastrointestinal conditions and sugar syrups, mutual antagonism between the LAB strains, hemolytic activity, mucin degradation, bile salt hydrolase (BSH) activity, and hydrogen peroxide production capacity. The conducted experiments facilitated the determination of the LAB probiotic potential. Furthermore, the cytotoxicity of the LAB metabolites at various concentrations was determined. The LAB strains were also tested for their ability to produce organic acids, also short-chain fatty acids (SCFAs) that have beneficial effects on host health. Finally, we investigated the protective activity of cell-free supernatants (CFS) from the LAB against two insecticides in Caco-2 cells, which is the novelty of the study. The results of our research suggest interesting prospects for using tested LAB to improve the viability of honeybees and the conditions in apiaries.

## 2. Materials and Methods

### 2.1. Bacterial Strains and Growth Conditions

Eleven strains of LAB were used for this study. Most of the strains have been isolated from the honeybee environment, i.e., flowers from which honeybees collect nectar and various kinds of honey. LAB used in the research were: *Pediococcus acilactici* 4/1 (from *Robinia pseudoaccacia* L. flowers), 11/3 (from *Philadelphus coronaries* L. flowers), 18/1 (from *Buddleja davidii* L. flowers) and 21/1 (from spoiled/fermented honey), *Pediococcus pentosaceus* 5/2 (from *Weigela florida* DC. flowers), 7/1 (from *Papaver rhoeas* L. flowers), 14/1 (*from Lavandula augustifolia* L. flowers), 25/1 (from heather-nectar honey) and OK-S (from fermented cucumbers), and *Lacticaseibacillus casei* 12AN (from human feces). Nine out of eleven LAB strains were genetically identified using 16S rRNA gene sequencing. The resulting sequences were deposited at the NCBI GenBank database under the following Accession Numbers: *P. acidilactici* 4/1 (OP598588.1); *P. pentosaceus* 5/2 (OP598591.1); *P. pentosaceus* 7/1 (OP598592.1); *P. acidilactici* 11/3 (OP598590.1); *P. pentosaceus* 14/1 (OP598589.1); *P. acidilactici* 18/1 (OP598587.1); *P. acidilactici* 21/1 (OP598586.1 and OP598803); *P. pentosaceus* 25/1 (OP598593.1), *and P. pentosaceus* OK-S (OP598594.1 and OP598810.1). All identified strains were deposited in the collection of the Department of Environmental Biotechnology, Lodz University of Technology. *L. casei* 12AN was from the above-mentioned collection and has been identified previously (unpublished data). Additionally, *Apilactobacillus kunkeei* DSM 12361 (from honeybee gut), was used as a reference (control) strain and it was purchased from the German Collection of Microorganisms and Cell Cultures GmbH.

*Staphylococcus aureus* ATCC 6538 and *Salmonella enterica* subsp. *enterica* serovar Typhimurium ATCC 14028 were used as positive controls in selected experiments (hemolysis and mucin degradation assay, accordingly). All bacteria were stored in Cryobanks™ at −20 °C. Before the start of the experiments they were activated, threefold passaged (3% inoculum), and cultured with AnaeroGen Atmosphere Generation Systems sachets in MRS medium for 24 h at 37 °C. *A. kunkeei* DSM 12361 was anaerobically cultured on MRS broth with the addition of fructose (10 g/L) and 0.05% cysteine-hydrochloride (MRS-F). *S. aureus* ATCC 6538 and *S*. Typhimurium ATCC 14028 were cultured on TSB and TSA for 24 h at 37 °C.

A list of culture vessels, chemicals, and other materials used in the research is presented in Appendix A.

### 2.2. High-Performance Liquid Chromatography (HPLC)

The quantification of organic acids profiles in CFS was performed according to the method presented by Chen et al., with some modifications [30]. MRS/MRS-F broth was inoculated with an individual strain of LAB and then incubated for 24 h at 37 °C. The samples were centrifuged (10,733× *g*, 15 min); next, CFS were filtered with sterile syringe filters (0.22 μm) and frozen until analysis at −20 °C. The physiological pH of CFS was from 3.81 (for *P. pentosaceus* 25/1) to 4.51 (for *A. kunkeei* DSM 12361), depending on the strain. Determination of lactic, malonic, acetic, butyric, and propionic acids was conducted using HPLC. Acids were analyzed qualitatively and quantitatively by comparing standard solutions with CFS from LAB. The chromatographic separation, absorbance measurement, and calculation of organic acid concentrations were determined according to the methodology presented in our previous publication [31].

### 2.3. Survival in the Simulated Digestive Tract

The study was performed in laboratory conditions on the basis of existing literature that used various models of the simulated digestive tract [32,33,34,35]. Since there is no such developed model for the honeybee, the composition of the simulated gastric and intestinal juices was slightly modified to match the conditions prevailing in the honeybee’s digestive system. Overnight LAB cultures in MRS/MRS-F broth were centrifuged (10,733× *g,* 15 min), decanted, and suspended in sterile 0.85% NaCl. This operation was repeated twice until the culture medium was completely rinsed off. The LAB biomass was suspended in fresh simulated gastric juice (containing 0.15% pepsin, 100 U/mL α-amylase, 0.85% NaCl, pH adjusted to 3.0, sterile filtered with 0.22 µm pore size syringe filters) and in 0.85% NaCl (negative control) and incubated for 2 h at 35 °C with shaking (80 r.p.m.). Next, the fresh simulated intestinal juice (containing 1% bile salts, 0.1% pancreatin, 0.85% NaCl, pH adjusted to 7.5, sterile filtered with 0.22 µm pore size syringe filters) was added to each test sample, and incubation was continued for a further 2 h. At each time point (initial 0 h, after 2 h incubation in simulated gastric juice, and after further 2 h incubation in simulated intestinal juice) 1 mL of each sample was transferred to sterile 0.85% NaCl, mixed, diluted, plated on agar plates with MRS/MRS-F according to the Koch plate method, and after 48 h incubation, the colonies growing on agar were counted.

### 2.4. Survival in the Sugar Syrups

The methodology was performed on the basis of existing literature data [36,37,38] with some modifications. Additionally, the composition of sugar syrups simulated the content of syrups applied in beekeeping. The first steps were conducted exactly as described in the case of Section 2.3. Next, The LAB biomass of each strain was suspended in syrup A (40% glucose, 30% fructose, pH 4.2, distilled water), syrup B (30% glucose, 40% fructose, 30% saccharose, distilled water), and syrup C (50% saccharose, distilled water). The pH of each syrup was adjusted to 4.2, and all were sterile filtered with 0.22 µm pore size syringe filters. The negative control was LAB biomass suspended in sterile distilled water. The samples were incubated for 24 h and 48 h at 20 °C. After this double incubation, 1 mL of each sample was transferred to sterile 0.85% NaCl, mixed, diluted, plated on agar plates with MRS/MRS-F according to the Koch plate method, and after 48–72 h incubation the colonies growing on agar were counted.

### 2.5. Cytotoxicity of CFS

#### 2.5.1. Caco-2 Cell Culture

Caco-2 (human colon adenocarcinoma) cells were cultured in high-glucose DMEM, 10% FBS, 4 mM GlutaMAX^TM^, 25 mM HEPES, and streptomycin (100 µg/mL)/penicillin (100 IU/mL) mixture as a monolayer (37 °C, 5% CO_2_) in a humidified incubator (Galaxy 48S, New Brunswick, United Kingdom) for 7 days, to reach 80% confluence. Every 2–3 days, the cells were washed with PBS (pH 7.2) without calcium and magnesium, and the medium was renewed. Confluent cells were detached from the culture with TrypLE^TM^ Express (37 °C, 8 min), centrifuged (307× *g*, 5 min), decanted, and then the pellet was resuspended in a fresh culture medium. Cell viability was determined by trypan blue exclusion and a cell count in a hemocytometer. The viability of cells was 95%.

#### 2.5.2. Neutral Red Uptake (NRU) Assay

The cytotoxicity was assessed with NRU assay. A total of 10,000 Caco-2 cells per well were seeded into a 96-well flat-bottom transparent plate and incubated for 24 h at 37 °C in 5% CO_2_. The next day, the medium was aspirated from the cells monolayer, and then CFS at physiological pH (in 4 replicates) were added at the following final concentrations [mg/mL]: 10, 50, 100, and 200 (*v/v*). The exposition time was 24 h. Negative controls were non-exposed cells in culture DMEM. Next, tested samples were aspirated, NR (50 μg/mL in PBS) was added to each well, and plates were incubated for 3 h. Then, the NR solution was aspirated and extracted from the cells with a desorbing solution (1% acetic acid, 50% ethanol, and 49% distilled water). The absorbance measurement was carried out in accordance with the methodology published by us previously [39]. Two independent experiments were conducted. Results were presented as the mean of the two independent experiments ± standard deviation (±SD).

#### 2.5.3. Protective Activity of CFS

This stage of the study used CFS (the pH adjusted to 7.0 ± 0.1 with 0.1 M NaOH) of *P. pentosaceus* 14/1. This strain was selected based on our previous studies [28]. Caco-2 cells were seeded into a 96-well plate at the amount of 10,000 cells per well and incubated for 24 h at 37 °C in 5% CO_2_. Next, the medium was aspirated and cells were exposed to 0.1 and 1 mg/mL (IC_0_) of CFS of *P. pentosaceus* 14/1 for 24 h (in 4 replicates). After that time, CFS were aspirated, cells were gently washed with PBS and the following insecticides concentrations were added to wells on cell monolayer: chlorpyrifos (25 and 50 µg/mL), and coumaphos (12.5 and 25 µg/mL), which were ≤IC_50_. Insecticide stocks were dissolved in sterile DMSO, so the final concentration of DMSO in the experiment was nontoxic for cells (≤0.5%). Then, the cells were incubated for a further 24 h at 37 °C in 5% CO_2_. IC_0_ of CFS and IC_50_ of insecticides were established earlier in screening tests (unpublished data). Negative controls were non-exposed cells in DMEM. After that time, cytotoxicity was measured as described in Section 2.5.2.

### 2.6. Antibiotics Susceptibility Testing

The susceptibility of LAB to antibiotics was tested using the disc diffusion method. The following antibiotics were investigated: ampicillin (10 µg), chloramphenicol (30 and 50 µg), enrofloxacin (5 µg), erythromycin (30 µg), gentamycin (30 µg), kanamycin (30 µg), lincomycin (2 and 15 µg), oxytetracycline (30 µg), streptomycin (25 µg), sulfonamides (300 µg), tetracycline (30 µg), and vancomycin (30 µg). Overnight cultures of LAB at the density of 1.8 × 10^9^ CFU/mL (6.0 according to McFarland Standard) were applied with a sterile disposable spatula onto Petri dishes with MRS/MRS-F agar. Then, paper discs impregnated with antibiotics were placed in triplicate for one plate and incubated at 37 °C for 24 h. In the case of tylosin, sterile paper discs were impregnated with the antibiotic at a concentration of 30 µg. After the plates were incubated, the diameter of the zone of inhibition of microbial growth was observed and measured in millimeters. Interpretation of the antibiotic susceptibility profile of LAB was performed according to previous publications [40,41,42,43,44,45,46,47]. The susceptibility was expressed in terms of susceptible (S), moderately susceptible (MS), and resistant (R).

### 2.7. Hydrogen Peroxide Production

The ability of LAB to produce H_2_O_2_ was evaluated on the basis of Song et al. [48]. Overnight cultures of LAB were streak plated on agar MRS/MRS-F supplemented with 0.25 mg/mL TMB and 0.01 mg/mL horseradish peroxidase and incubated for 48 h at 37 °C. A blue color of the colony indicates a positive test result.

### 2.8. Hemolysis

The experiment was conducted on the basis of Yasmin et al. [49] and de Albuquerque et al. [50] with the streak plate method. Each LAB strain was inoculated on the surface of MRS/MRS-F agar with 5% of defibrinated horse blood and incubated for 24–48 h at 37 °C. According to Wang et al., *S*. *aureus* exhibits various hemolytic activities that can damage host cell membranes [51]. In our study, the positive control was *S. aureus* ATCC 6538 correspondingly growing on TSA. The assay was conducted for two repeats for each strain. The presence of hemolysis was assessed as follows: greenish zones under and around the colonies (α-hemolysis); clear zones under and around the colonies (β-hemolysis); no zones around colonies (γ-hemolysis—i.e., no hemolysis).

### 2.9. Mucin Degradation

The experiment was conducted on the basis of Yasmin et al. [49] and de Albuquerque et al. [50] with the streak plate method. Each LAB strain was inoculated on the surface of MRS/MRS-F agar supplemented with 0.5% of mucin from the porcine stomach with 3% or without glucose and incubated for 72 h at 37 °C. Then, the agar plates were stained with 2% (*w*/*v*) malachite green in 3.5 M acetic acid for 30 min and washed with 1.2 M acetic acid. Mucin degradation was considered positive when a clear zone surrounding the colonies was observed. The assay was conducted for two repeats for each strain. The positive control was *Salmonella typhimurium* ATCC 14028 correspondingly growing on TSA with 0.5% off mucin.

### 2.10. Bile Salts Hydrolase (BSH) Activity

The experiment was conducted on the basis of de Albuquerque et al. [50]. Overnight cultures of LAB were streak plated on agar MRS/MRS-F supplemented with 0.5% taurodeoxycholic or 0.5% glicodeoxycholic acid and incubated for 5 days at 37 °C. Opaque zones around the colony testify to bile salt deconjugation.

### 2.11. Mutual Antagonistic Activity with Agar Slab Method

LAB at the density of 1.8 × 10^9^ CFU/mL (6.0 according to McFarland Standard) were applied with a sterile disposable spatula onto Petri dishes with MRS/MRS-F agar and incubated for 24 h at 37 °C. The disks (10 mm diameter) were cut in triplicate from the solid medium and placed on MRS medium containing different LAB strain at the density of 6.0 × 10^8^ CFU/mL (2.0 according to McFarland Standard). Subsequently, plates were incubated for 24 h at 37 °C.

### 2.12. Statistical Analysis

The results in Table S1 (survival of LAB strains in the simulated digestive tract), Table S2 (survival of LAB strains in sugar syrups), Tables S3 and S4 (survival of Caco-2 cells in the presence of CFS of LAB strains), and Table S5 (protection activity of CFS against the cytotoxicity of insecticides) are presented as the mean from three/four repeats ± standard deviation (SD).

Non-parametric tests were used for statistical analyses of the analyzed parameters as survival of LAB strains in the simulated digestive tract, in sugar syrups, and protection against the cytotoxicity of insecticides did not follow a normal distribution (Shapiro–Wilk test). Differences regarding the analyzed parameters were tested using the Kruskal–Walli’s test (KW test), followed by a multiple comparison test (MCT) to indicate significant differences between the groups. In order to compare the scores on the different LAB strains’ viability in the simulated digestive tract in comparison to the *A. kunkeei* DSM 12361 naturally inhabiting the honeybee intestines, the Independent Samples *t* Tests (*t*-Test) were used.

A *p*-value < 0.05 was considered statistically significant. The KW, MCT, and Independent Samples *t* Tests were performed using Statistica ver. 13.1 (StatSoft, Tulsa, OK, USA).

## 3. Results and Discussion

### 3.1. Organic Acids Profile

**Figure 1:**
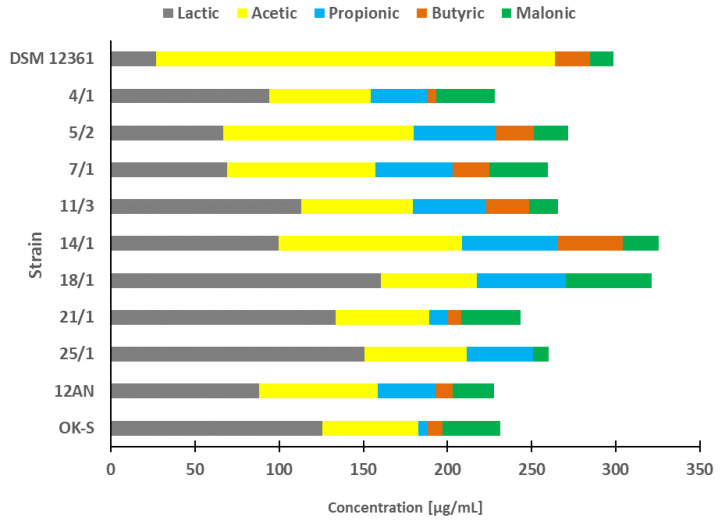
Organic acids profiles in cell-free supernatants from lactic acid bacteria after 24 h cultivation in MRS medium.

In our study, we evaluated the acid profiles of 10 LAB strains isolated from different environments (Figure 1). In previous publications, these strains demonstrated the most potent antagonistic activity against honeybee pathogens (e.g., *Paenibacillus* spp., *Melissococcus plutonius*), insecticide detoxification capacity, and adhesion ability to various surfaces [26,27,28]. Additionally, *A. kunkeei* DSM12361 isolated from the honeybee gut microbiota was used as a reference strain. The acid concentrations detected varied depending on the strain tested. The bacterial isolates that produced the highest amounts of all acids combined (over 300 µg/mL) were *A. kunkeei* DSM 12361, *P. pentosaceus* 14/1, and *P. acidilactici* 18/1. All LAB strains produced lactic, acetic, and malonic acids. Butyric acid was not detected only in CFS from *P. acidilactici* 18/1 and *P. pentosaceus* 25/1, however, *P. acidilactici* 18/1 produced the highest concentrations of lactic acid among all tested isolates.

Organic acids perform an antimicrobial function in the host organism and reduce the risk of infections caused by various pathogens [52]. Lactic acid strengthens the gut barrier and promotes bowel regularity in the host’s digestive tract [53,54]. In addition, this organic acid increases the absorption of compounds such as flavonoids that perform various antioxidant functions [55]. In in vitro studies by Ruiz Rodriguez et al., lactic acid was the main fermentation end product with concentrations ranging between 0.6 and 4.9 g/l [56]. LAB strains that produced the highest concentrations of this acid were *L. lactis* subsp. *Lactis* FN3-317 and F-Cq1-484-2, *E. faecium* F30-P1-154 and *Leuconostoc pseudomesenteroides* Cq1-248 and Cq1-272 [56]. Gas chromatography-mass spectrometry performed by Lee et al. showed that the production of lactic acid by LAB strains (*Lactiplantibacillus pentosus* K34 and *Pediococcus lolii* PL24) was more abundant compared to the rest of the metabolites and the profile of organic acids differed depending on the tested isolate [57]. In our study, lactic and acetic acids were the dominant organic acids produced. The strain that produced significantly higher concentrations of acetic acid than the rest of the isolates was the fructophilic *A. kunkeei* DSM 12361. Acetic acid facilitates the digestion of food and can relieve constipation of the large intestine [58]. The decomposition of pyruvic acid in the heterolactic fermentation pathway can lead to the production of acetic acid in the addition to lactic acid [10]. Fructophilic LAB (including strains of the species *A. kunkeei*) are heterofermentative microorganisms that use fructose as the main carbon source and produce both of the above-mentioned acids [59]. The organic acid profile of *A. kunkeei* DSM 12361 presented in our study coincides with the results of in vitro tests conducted by Filannino et al. [60]. Acetic acid reached higher concentrations compared to the rest of the end-products (lactic acids, ethanol, and mannitol), ranging from 40.1 mM/L for *F. fructosus* to 66.2 mM/L for *A. kunkeei* B4I [60].

Other SCFAs detected in the CFS we tested were propionic and butyric acids. The highest concentrations of these acids were detected in CFS from *P. pentosaceus* 14/1, which in our previous study showed potent protective activity against chlorpyrifos, coumaphos, and imidacloprid [28]. Butyric acid and its derivatives exhibit anticancer properties, which may be related to the ability of *P. pentosaceus* 14/1 to reduce the cytotoxicity and genotoxicity of insecticides [28,61,62]. The only strain that did not produce propionic acid was *A. kunkeei* DSM 12361. It has been suggested that propionic acid improves tissue insulin sensitivity [63]. Furthermore, propionic acid has an effect on immunosupportive actions and lowers fatty acid levels in the plasma and liver [63]. Butyric acid contributes to anti-inflammatory activity and regulates microbiota composition and gastrointestinal motility [64]. According to Khalil et al., LAB isolated from functional Malaysian food exhibited different capacities to produce organic acids [65]. Similar to the results of our study, propionic and butyric acids were produced in lower concentrations compared to acetic and lactic acid [65]. In our study, we also demonstrated that all tested LAB strains produced malonic acid belonging to the group of dicarboxylic acids. Malonic acid can reduce collagen damage and inhibit inflammatory factor activity [66]. Contrary to our experiment, Seo et al. demonstrated that *L. brevis* Wi-Kim0069 isolated from kimchi did not exhibit the capacity to produce this acid [67].

### 3.2. Ability of LAB Strains to Survive in the Simulated Digestive Tract

**Figure 2:**
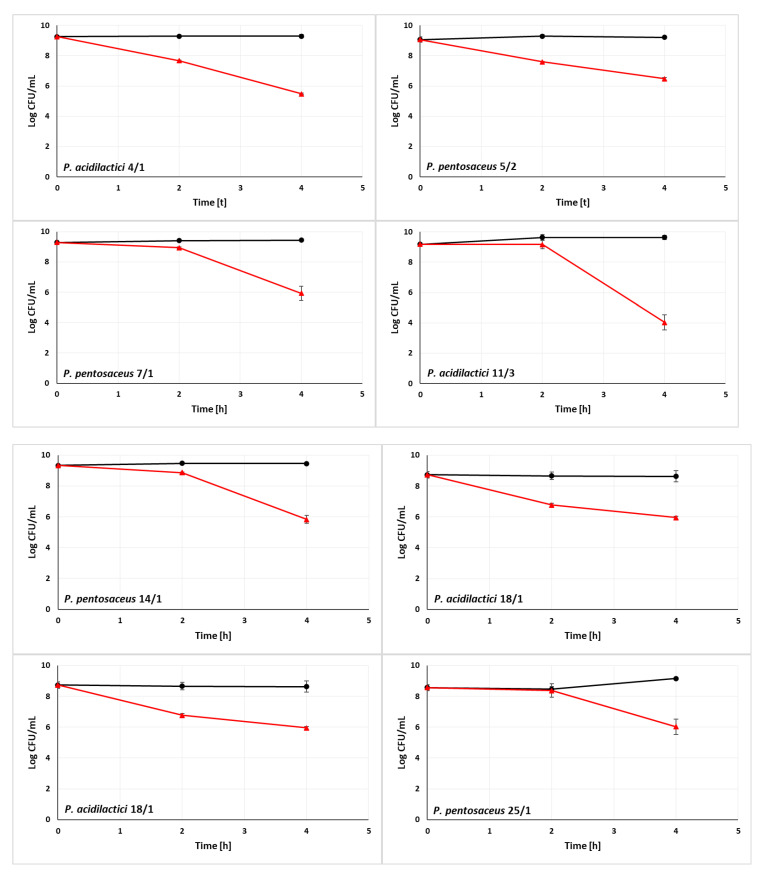

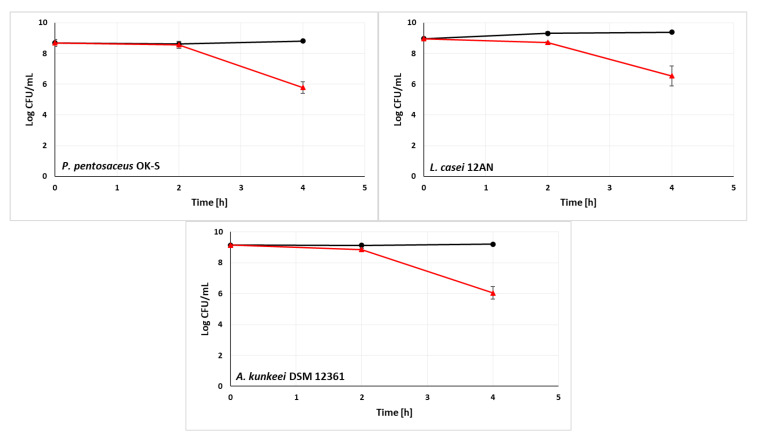
Resistance of lactic acid bacteria (LAB) strains to gastric (*t* < 2 h) and intestinal (*t* > 2 h) conditions. The black line (square markers) indicates pure LAB culture (control) and the red line (triangular markers) indicates LAB incubated with gastric and intestinal juices. Results are presented as mean ± standard deviation.

In in vitro studies, the viability of LAB at low environmental pH and in the presence of bile salts or in a simulated gastrointestinal tract are key characteristics of the bacteria to be considered probiotics [68,69]. In our study, we evaluated the resistance of 10 LAB strains to a simulated digestive tract (Figure 2). *A. kunkeei* DSM 12361 which naturally inhabits honeybee gut was used as a reference strain. The composition of gastric and intestinal juices is described in Section 2.3. In the presence of simulated gastric juice, most strains showed a high survival rate on a comparable level to the *A. kunkeei* DSM 12361. The viability of *P. acidilactici* 4/1 and 18/1, and *P. pentosaceus* 5/2 was strongly decreased and the *P. acidilactici* 11/3 was significantly increased in comparison to the reference strain (*p* = 0.0008, *p* = 0.0007, *p* = 0.0006, *p* = 0.049 respectively, Independent Samples *t* Tests) (Table S1 in Supplementary Materials). This suggests the resistance of isolates to the mixture of pepsin, α-amylase, and the acidic pH of the environment. LAB strains displayed high viability after 2 h of incubation in the simulated gastric environment, which coincides with the recognized values of acid tolerance of probiotic microorganisms [70]. An increase in LAB mortality was noticed after incubation in simulated intestinal juice. The increased bacterial mortality may have been caused by the presence of pancreatin and bile salts. However, all isolates presented a viable cell count above 10^4^ CFU/mL with a recognized detection limit of probiotic bile salt resistance of 10^3^ CFU/mL [33]. The survival rate of tested LAB was on a comparable level to the reference strain *A. kunkeei* DSM 12361, as there were no statistical differences in the LAB viability in the intestinal juice (*p* > 0.05, Independent Samples *t* Tests) (Table S1 in Supplementary Materials). This suggests the ability of isolates to colonize environments resembling the conditions prevailing in the digestive tract of a honeybee.

According to in vitro tests carried out by de Oliveira Coelho et al. 3 strains (*Liquorilactobacillus satsumensis* LPBF1, *Saccharomyces cerevisiae* LPBF3, and *L. mesenteroides* LPBF2) isolated from honey-based kefir exhibited tolerance to simulated gastric and intestinal conditions. The isolates showed a high survival rate in acidic and neutral pH of the environment in all tested times (1, 2, 3, and 4 h) [33]. LAB strains tested by Tokatli et al. showed higher viability in a gastric juice environment than in intestinal conditions [71]. The strains of the species *L. brevis* and *L. plantarum* exhibited the highest resistance to bile salts, and the demonstrated properties turned out to be strain-dependent features [71]. In order to be considered probiotic microorganisms, LAB should be resistant to low pH, pepsin, NaCl, and bile salts [72,73]. According to Mantzourani et al., 10 LAB isolates showed probiotic potential and resistance to acidic pH [74]. Strains SP5 and SP2 (later identified as *Lacticaseibacillus paracasei* and *P. pentosaceus*, respectively) achieved the highest viability similar to the survival value of the probiotic *L. plantarum* ATCC 14971 [74]. The strains exhibiting the highest resistance to low pH and bile salts in in vitro tests performed by Feng et al. were subjected to further research to determine the survival rate of isolates in gastrointestinal conditions [75]. Resistance to simulated gastric juice was strain-dependent and varied according to incubation time. The highest survival rate in gastric conditions was demonstrated *by E. faecium* WEI-9 and WEI-10 (above 97%). However, LAB resistance to small intestinal juice significantly differed between isolates [75]. In contrast to our study, 66 LAB strains (including 24 isolates from probiotic products) tested by Masco et al. showed a decrease in viability in the presence of pepsin [76]. The highest survival rate in gastric conditions was demonstrated by *B. animalis* subsp. *lactis* [76]. According to Benavides et al., LAB resistance to NaCl depends on salt concentration and incubation temperature [77]. The viability decrease of 11 LAB isolates was observed after incubation in 6% NaCl at both tested temperatures [77]. Furthermore, a factor that may affect the resistance of LAB to NaCl is the presence of chemical compounds such as ethanol and H_2_O_2_ [58]. Our study suggests that LAB strains demonstrating the highest viability in simulated gastrointestinal conditions can be used to construct a probiotic preparation for honeybees.

### 3.3. Ability of LAB Strains to Survive in the Sugar Syrups

**Figure 3:**
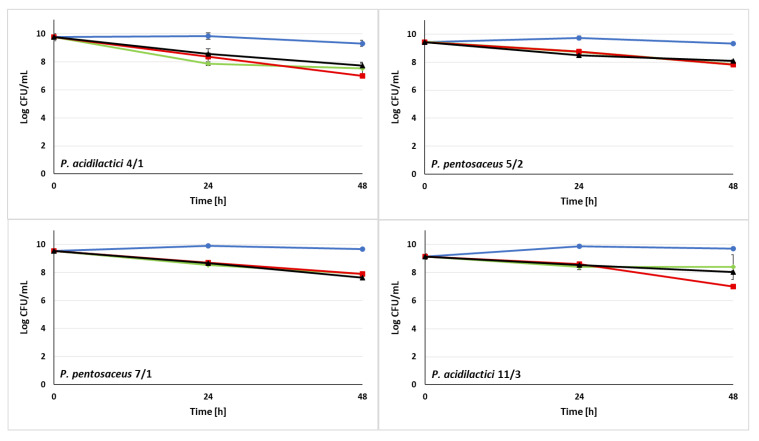

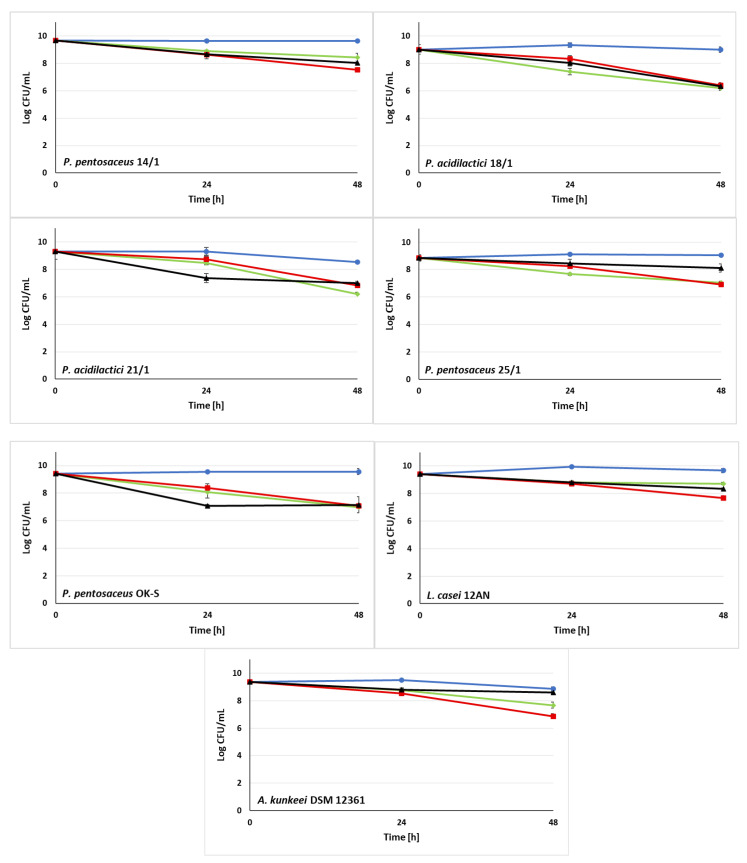
Survival of lactic acid bacteria (LAB) strains in sugar syrups A, B, and C after 24 and 48 h of incubation. The color of the line indicates the incubation conditions of LAB. Blue (circular markers)—no sugar syrup (control); green (diamond markers)—syrup A (40% glucose, 30% fructose, pH 4.2, distilled water); red (square markers)—syrup B (30% glucose, 40% fructose, 30% saccharose, distilled water); and black (triangular markers)—syrup C (50% saccharose, distilled water). Results are presented as true mean ± standard deviation.

Sugar syrups are often used as a nutrition source for honeybees [78]. Refined sugar syrups in the pre-winter period can be more suitable for feeding these pollinators than honey [78]. Furthermore, one of the methods of administering probiotic strains to honeybees is the supplementation of sugar syrups [79]. In order to perform protective functions in the organisms of honeybees, probiotic LAB candidates should demonstrate high resistance to the presence of various components of sugar syrups used in beekeeping. In the above study, we tested the viability of 10 LAB strains and a reference strain *A. kunkeei* DSM 12361 in the presence of 3 different sugar syrups (Figure 3). The compositions of the syrups are presented in Section 2.4. The tested isolates exhibited a varied and rather high survival rate. After 48 h of incubation, LAB demonstrated a viable cell count above 10^6^ CFU/mL in all tested syrups. LAB isolates displayed high viable cell densities after 24 and 48 h of incubation in Syrup A ranging from 7.40 log CFU/mL for *P. acidilactici* 18/1 to 8.91 log CFU/mL for *P. pentosaceus* 14/1 and from 6.19 log CFU/mL for *P. acidilactici* 18/1 to 8.72 log CFU/mL for *L. casei* 12AN, respectively. *P. pentosaceus* 5/2 and *P. acidilactici* 18/1 and 21/1 exhibited significantly decreased survival rates after 48 h incubation in syrup A (*p* = 0.017, *p* = 0.026, *p* = 0.008, respectively; KWW test) (Table S2 in the Supplementary Materials). The highest viability in Syrup B was demonstrated by *P. pentosaceus* 7/1, which viable cell concentration reached 7.91 log CFU/mL after 48 h of incubation. A significantly decreased bacterial survival rate in the presence of syrup B was observed for *A. kunkeei* DSM 12361, *P. acidilactici* 21/1, *P. pentosaceus* 25/1, *P. pentosaceus* 5/2, *P. acidilactici* 4/1, *P. acidilactici* 11/3, *P. pentosaceus* 14/1 and *L. casei* 12AN (*p* = 0.002, *p* = 0.023, *p* = 0.01, *p* = 0.0178, *p* = 0.008, *p* = 0.004, *p* = 0.002, *p* = 0.002, respectively). In the presence of Syrup C, the viable cell density of LAB was between 7.07 log CFU/mL for *P. pentosaceus* OK-S and 8.83 log CFU/mL for *L. casei* 12AN after 24 h of incubation and between 6.34 log CFU/mL for *P. acidilactici* 18/1 and 8.58 log CFU/mL for *A. kunkeei* DSM 12361 after 48 h of incubation. A significantly potent decrease in bacterial viability was demonstrated by *P. acidilactici* 18/1 and 21/1, and *P. pentosaceus* 7/1 (*p* = 0.008). All LAB strains exhibited high levels of osmotic tolerance in the presence of tested sugar syrups.

According to Iorizzo et al., 10 strains of *A. kunkeei* inhabiting the digestive tract of honeybees exhibited tolerance to high concentrations of sugars [36]. *A. kunkeei* DSM 12361, which was also used in our study, showed significantly lower viability compared to the rest of the LAB isolates after 48 h of incubation in syrup B (containing 30% fructose and 40% glucose) [36]. In vivo tests carried out by Pachla et al. presented reduced honeybee mortality after sucrose syrup supplementation with LAB [38]. *A. kunkeei* CH1 and *Fructobacillus fructosus* V5 and VIII1 significantly improved pollinator viability [38]. Furthermore, honeybees infected with pathogens such as *Nosema ceranae* exhibit a higher sugar syrup consumption rate [37]. It has been suggested that LAB affect the digestibility of sugars by contributing to the reduction of energy stress in the organisms of infected pollinators [37]. The resistance of LAB differed depending on the tested strain and syrup. The majority of tested LAB strains demonstrated significantly high resistance while incubated with syrups A and C (as a significantly decreased cell viability has been observed), offering promising prospects for improving the viability of honeybees and suggesting the possibility of administering probiotic candidates along with sugar syrups.

### 3.4. Cytotoxic Activity of CFS

**Figure 4:**
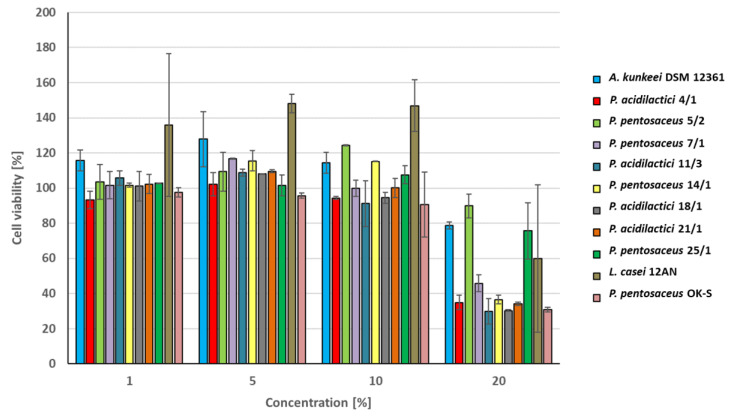
Survival of Caco-2 cells in the presence of cell-free culture supernatants from lactic acid bacteria (LAB). Results are presented as mean ± standard deviation. Results showing the statistically significant effect of metabolites produced by LAB on cell survival rate are presented in Tables S3 and S4 (*p* < 0.05).

In this study, we used the Caco-2 cell line which consists of human colorectal cancer cells and is often used as a model of the intestinal epithelial barrier [80]. The results of the research were presented as cell viability in the presence of CFS from LAB (Figure 4). Results showing the statistically significant effect of LAB metabolites on cell survival rate are presented in Tables S3 and S4 (*p* < 0.05). Caco-2 cells showed high viability in the presence of CFS at concentrations of 1, 5, and 10%. Cytotoxicity was observed only for the highest concentration of bacterial metabolites (20%). The highest cell survival at this concentration was noted for CFS from *P. pentosaceus* 5/2 (89.81% ± 8.74%) and 25/1 (75.65% ± 15.2%). The metabolites of these strains demonstrated similar (or lower) cytotoxicity in comparison to *A. kunkeei* DSM 12361 (78.72% ± 6.66%) used in this study as a reference strain isolated from the digestive tract of a honeybee. This suggests that the tested CFS display a relatively comparable effect on the Caco-2 cell line to *A. kunkeei* DSM 12361 and do not threaten the viability of cells found in organisms of these pollinators. LAB produce postbiotic metabolites (e.g., organic acids, SCFAs, exopolysaccharides, enzymes, and cell wall fragments) that perform various probiotic functions in the host body [81,82]. The result of the 3-(4,5-dimethylthiazol-2-yl)-2,5-diphenyltetrazolium bromide (MTT) assay conducted by Avand et al. showed that nisin produced by *Lactococcus lactis* subsp. *Lactis* PTCC 1336 exhibits selective and high cytotoxicity against a breast cancer cell line (MCF-7) with an IC_50_ value of 5 μM [83]. Moreover, significantly higher cytotoxicity was observed after the combination of nisin with doxorubicin, suggesting a synergistic effect of these chemicals on cell viability [83]. According to Nowak et al., the cytotoxicity of the LAB post-fermentation media depended on their concentration and reached higher values than the cytotoxicity demonstrated by the cell extracts, which also strongly affected the viability of Caco-2 cells [31]. Chuah et al. determined the selective cytotoxic effect of postbiotic LAB metabolites produced by 6 strains of *L. plantarum* on various cancer cell lines [84]. The strongest cytotoxic effect on human breast cancer cell MCF-7 viability was noted for postbiotics from *L. plantarum* UL4PM. In comparison, metabolites of *L. plantarum* RG14 displayed the highest cytotoxicity toward leukemia cells HL60 [84]. The tested postbiotics produced by 6 LAB strains showed various cytotoxic effects on cancer cells, without affecting the viability of nonmalignant MCF-10A cells used as a reference [84]. The cytotoxicity of compounds produced by LAB depends on the tested strain, cell line, and concentration of CFS. The effect of LAB metabolites on cell viability in honeybee organisms should be also evaluated in additional in vivo tests to confirm the safety of probiotic candidates.

### 3.5. Protective Activity of CFS of P. pentosaceus 14/1 against Insecticides

**Figure 5:**
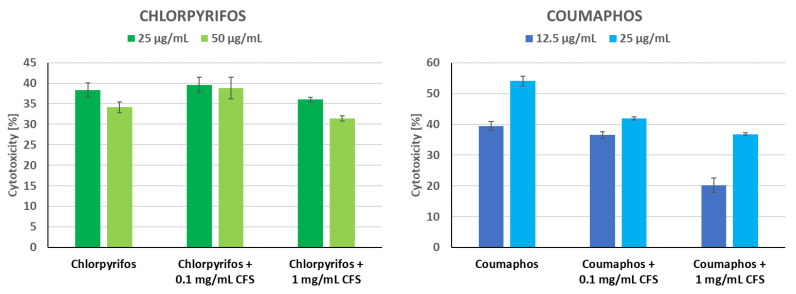
The ability of *P. pentosaceus* 14/1 cell-free supernatant to protect Caco-2 cells against the cytotoxicity of insecticides. Firstly cells were exposed to cell-free supernatant at IC_0_ (24 h, unpublished data) and then to selected insecticide at concentration ≤ IC_50_ (24 h). Results are presented as mean ± standard deviation.

In the above study, we determined the effect of CFS from *P. pentosaceus* 14/1 on the reduction of insecticide cytotoxicity (Figure 5). This strain was selected because of the strong binding capacity of these insecticides to the cell wall of up to 64% as published previously [28]. The strain also exhibited high viability in the presence of insecticides [28]. In the current study, the Caco-2 cell line was used due to the lack of a commercially available continuous cell line derived from honeybees. Evaluation of insecticide detoxification capacity of LAB metabolites in human intestinal Caco-2 cell line is significant considering the fact that insecticides can be ingested by humans along with contaminated honeybee products. Caco-2 cells showed similar sensitivity to chlorpyrifos and coumaphos (Figure 5). In agriculture, coumaphos is used to control livestock pests and insects [85]. This organophosphate-based acaricide affects the detoxification functions and immune responses of honeybees, contributing to increased colony mortality [86]. In our study, CFS at a concentration of 1 mg/mL showed the most potent detoxification of coumaphos. After incubation with LAB metabolites, the cytotoxicity of this insecticide at concentrations of 12.5 and 25 µg/mL decreased by 19.32% and 17.23%, respectively. After incubation with 1.0 mg/mL CFS, the toxicity of coumaphos at a concentration of 25 µg/mL was significantly decreased (*p* = 0.003, KW test) (Table S5 in the Supplementary Materials). This suggests the effect of CFS on detoxifying toxic substances and increasing cell viability. LAB metabolites at a concentration of 0.1 mg/mL slightly reduced the cytotoxicity of chlorpyrifos. Chlorpyrifos is an organophosphorus insecticide that threatens the health of honeybees for up to 3 days after application to crops [87]. Exposure to this pesticide increases the mortality of honeybee larvae and causes learning diseases in adult individuals [88]. Zhou and Li evaluated the cytotoxicity of chlorpyrifos to the HepG2 cell line [75]. Chlorpyrifos exposure decreased cell viability, induced morphological changes in the cell nucleus, and reduced the mitochondrial transmembrane potential of cells [89]. An in vitro study carried out by Oostingh et al. demonstrated the cytotoxic effect of chlorpyrifos at concentrations ≥250 µM [90]. The different results obtained after determining the cytotoxicity of diazinon suggested that cell viability is dependent on the pesticide tested [90]. Chlorpyrifos exposure has a strong cytotoxic effect on *Lens culinaris* apical cells and induces chromosomal abnormalities such as chromosome disruption, nucleus absence, irregular anaphase, micronuclei, and anaphase bridges [91]. Some chemical compounds exhibiting antioxidant properties can decrease the cytotoxicity of chlorpyrifos and reduce the inhibition of catalase and glutathione peroxidase activity caused by this insecticide [92]. In a previous study, we observed decreased cytotoxicity of three insecticides (chlorpyrifos, coumaphos, and imidacloprid) in the presence of potentially probiotic LAB strains [28]. The degree of detoxification depended on the tested strain, insecticide, and cell line, and the most potent cytotoxic effects were noted for *Spodoptera frugiperda* (Sf-9) cells [28]. Cho et al. studied the biodegradation of chlorpyrifos by LAB isolated during kimchi fermentation [93]. *L. brevis* WCP902, *Latilactobacillus sakei* WCP904, *L. plantarum* WCP931, and *L. mesenteroides* WCP907 were able to utilize this pesticide as a sole source of phosphorus and carbon [93]. In addition, the above-mentioned LAB strains also degraded coumaphos, parathion, diazinon, and methylparathion [93]. The results of the MTT assay performed by Bagherpour Shamloo et al. indicated that CFS from *L. casei* YSH isolated from traditional yogurt reduced the cytotoxic effects of diazinon on the human umbilical vein endothelial cells (HUEVEC) [94]. LAB metabolites at a concentration of 50 ug/mL significantly increased the cell viability in comparison to the control [94]. According to the authors’ knowledge, the effect of CFS from LAB on the cytotoxicity of insecticides has not yet been thoroughly investigated. Detoxification of pesticides by LAB metabolites is an important feature due to the negative effects of the honeybee microbiota dysbiosis, which include physiological and metabolic changes, weakening of resistance to infections caused by pathogens, and disruption of the detoxification system [95].

### 3.6. Susceptibility to Antibiotics

In our study, we tested the resistance of 11 LAB strains to 15 antibiotics (Table 1). LAB were selected based on their properties that we evaluated in our previous studies (e.g., antagonistic activity, detoxification of pesticides, and adhesive abilities) [26,27,28]. The diameter of the bacterial growth inhibition zone depended on the tested antibiotic and its concentration. Most of the tested LAB strains showed similar resistance to *A. kunkeei* DSM 12361 isolated from the digestive tract of honeybees and used in this experiment as a reference strain. The bacterial growth inhibition zone was not observed for enrofloxacin, gentamicin, kanamycin, streptomycin, sulfonamides, tylosin, and vancomycin. The greatest resistance to antibiotics was shown by *P. acidilactici* 4/1, which growth was also not inhibited by lincomycin. The strongest inhibition of LAB growth was observed for chloramphenicol. Antibiotic susceptibility profile was different between most bacterial strains (Table 1). Comparing the obtained results, it can be suggested that antibiotic resistance is a strain-dependent feature. LAB are recognized as carriers of antibiotic-resistance genes that can be propagated within the food chain between the environment, food, and the host organism [35]. Probiotics can restore the host microbiota after antibiotic treatment through endogenous resistance. LAB can also acquire resistance to antibiotics through horizontal gene transfer or gene mutation [35]. The antibiotics we tested can be divided into three categories due to their mechanism of action: inhibition of the cell wall synthesis (ampicillin and vancomycin), inhibitors of p-aminobenzoic acid in the folic acid metabolism cycle (sulfonamides), and inhibition of protein synthesis (chloramphenicol, enrofloxacin, erythromycin, gentamicin, kanamycin, lincomycin, oxytetracycline, streptomycin, tetracycline, and tylosin) [96,97]. The resistance demonstrated by LAB suggested no correlation between the antibiotic action mechanism and a probiotic candidate’s growth inhibition. LAB strains showed different spectra of resistance to kanamycin and ampicillin despite similar modes of action of these compounds. Erginkaya et al. tested the antibiotic resistance of 72 LAB isolated from dairy products [98]. The resistance profile showed that none of the isolates were resistant to ampicillin and nitrofurantoin. On the other hand, 58% of strains belonging to *Lactobacillus* spp. were resistant to vancomycin. LAB belonging to this genus exhibited sensitivity to chloramphenicol [98]. According to Amiranashvili et al., LAB isolated from the chicken gastrointestinal tract demonstrated varying spectra of susceptibility to 17 antibiotics [99]. All bacterial isolates were susceptible to tylosin, ampicillin, bacitracin, and rifampicin and resistant to nystatin, bacitracin, and metronidazole. LAB strains showed varying susceptibility to the other tested antibiotics [99]. In vitro tests carried out by Shazali et al. showed similarities in the antibiotic resistance profiles of *Latilactobacillus curvatus* and *Levilactobacillus brevis* [100]. *Lactobacillus acidophilus* was resistant to gentamycin, kanamycin, ciprofloxacin, vancomycin, sulphamethoxazole, streptomycin, and nalidixic acid. However, 7 species of LAB (*Ligilactobacillus salivarius*, *Limosilactobacillus fermentum*, *Lactobacillus delbrueckii* subsp *delbrueckii*, *P. pentosaceus*, *Lacticaseibacillus rhamnosus*, *Pediococcus damnosus*, and *Leuconostoc mesenteroides*) demonstrated sensitivity to all tested antibiotics [100]. The susceptibility of LAB to chloramphenicol demonstrated in our study was similar to the results presented by Somashekaraiah et al. [101]. Of all tested strains, only *Enterococcus durans* MYSN 10 and *Enterococcus faecium* MYSN 18 showed resistance to this antibiotic [101]. Antibiotics can have a negative effect on the health of honeybees [102]. For example, exposure to antibiotics can result in dysbiosis in the honeybee digestive tract by affecting the composition and size of the microbiome [103]. According to Ortiz-Alvarado et al., tylosin and oxytocin used frequently in beekeeping change the rate of behavioral development and the amount of lipids in the organisms of these pollinators [4]. Another antibiotic that threatens the health of honeybees is tetracycline, which high concentrations disturb the histological composition of cells in the digestive tract of the larvae [104]. In our study, the three above-mentioned antibiotics had the weakest inhibitory effect on the growth of *L. casei* 12AN, which was resistant to tylosin and showed intermediate susceptibility to tetracycline and oxytetracycline. The results of this research offer interesting perspectives on the construction of a preparation that can contribute to the improvement of the viability of honeybee colonies. The use of several tested strains in one preparation can expand the spectrum of antibiotic resistance due to the unique properties of the isolates.

### 3.7. Mutual Antagonistic Activity between LAB Strains

The tests were conducted to assess whether the LAB display mutual antagonism and thus whether they are suitable for a biopreparation as a mixture. None of the tested LAB strains showed mutual antagonistic activity, and no zones of growth inhibition were observed. The lack of growth inhibition of isolates has promising prospects considering the antagonistic abilities of these bacteria [27]. Therefore, it can be concluded that these strains will be able to be included in the same probiotic preparation for the honeybee. According to the authors’ knowledge, there are no in-depth studies on mutual antagonistic activity between LAB strains.

### 3.8. Hydrogen Peroxide Production, Hemolytic and BSH Activity, and Mucin Degradation

None of the LAB strains tested produced detectable hydrogen peroxide. No blue color of the colony was observed. The metabolic properties of LAB play an important role in antagonistic activity against pathogens [105]. The production of hydrogen peroxide by LAB can inhibit the growth of various pathogenic microorganisms [105]. Exposure to oxygen and/or light can contribute to H_2_O_2_ generativity by bacteria. Olofsson et al. investigated the H_2_O_2_ production ability of LAB isolated from the honeybee digestive system [106]. After in vitro testing, 5 out of 13 LAB strains were able to generate H_2_O_2_ in MRS media supplemented with TMB. Similar to the results of our study, the strain of genus *A. kunkeei* did not demonstrate the H_2_O_2_ production ability [106]. Wilks et al. studied H_2_O_2_ generativity by 73 LAB strains isolated from the vaginal swabs of pregnant women at high risk of preterm birth [107]. H_2_O_2_ production was a strain-dependent feature and the amount of compound produced varied between the LAB species tested [107]. Similar conclusions were presented by Mosbah and Mesbah [108]. The H_2_O_2_ production capacity of LAB also depends on the time of the experiment and the culture medium used [108,109]. According to Rabe and Killier, H_2_O_2_ production increased to 79% for TMB-Plus from 71% for TMB [109]. LAB strains grown on MRS or Rogosa medium supplemented with chromogen and peroxidase showed a lower ability to generate H_2_O_2_ [109]. In previous studies, we have shown that the LAB strains (also tested in this experiment) inhibited the growth of various pathogens [27]. The ability of probiotic candidates to produce H_2_O_2_ should be further confirmed by a more sensitive and reliable method, e.g., a semiquantitative assay [107].

None of the tested LAB strains showed hemolytic properties. After administration of the preparation containing LAB to honeybees, these bacterial isolates can be found in honeybee products consumed by humans and animals. Evaluation of the hemolytic activity of selected LAB was significant in terms of human safety. *S. aureus* ATCC 6538 used as positive control displayed β-hemolytic activity. Hemolysis of red blood cells exhibited by some pathogens may be related to the action of protein exotoxins, which contribute to the pathogenesis of infection [110]. In our study, we used *S*. *aureus* as a reference strain, which produces hemolysins that contribute to β-hemolysis and are significant virulence factors of this pathogen [111]. Similar results to our study were reported in an in vitro study carried out by Halder et al. [112]. The tested LAB strains (*Ligilactobacillus animalis* LMEM6, *L. plantarum* LMEM7, *L. acidophilus* LMEM8, and *L. rhamnosus* LMEM9) showed no β-hemolytic activity and no formation of clear zones associated with hemolysis was noted on the blood agar plates [112]. The safety evaluation of 5 LAB strains (*L. salivarius* M2-71, *E. durans* M2-3, *Limosilactobacillus mucosae* M4-7, *Enterococcus hirae* M5-8 and *E. faecium* M6-29) demonstrated no β-hemolysis of blood cells [113]. In contrast to our results, Benitez-Cabello showed β-hemolytic activity exhibited by LAB strains isolated from table olive biofilms and identified as *L. plantarum* [114]. The lack of recorded hemolysis of blood cells proves the safety of probiotic candidates for application in the organisms of honeybees.

None of the tested LAB strains showed mucin degradation properties. Pathogenic *S*. Typhimurium ATCC 14028 used as positive control demonstrated the degradation ability of mucin. Many epithelial surfaces (including intestinal cells) are covered with mucus, which is mostly composed of mucins. Mucins are glycoproteins, a component of bile and saliva, also found in the stomach and intestine, where they protect mucous membranes against the action of digestive enzymes [115]. Some LAB strains produce enzymes that contribute to the prevention of mucin degradation [116]. Zhou et al. evaluated the ability to degrade mucins by 3 potential probiotic strains (*Bifidobacterium lactis* (HN019), *L. rhamnosus* (HN001)*,* and *L. acidophilus* (HN017)) [117]. Similar to the results of our study, none of the LAB isolates degraded gastrointestinal mucins suggesting non-toxicity of the tested strains [117]. Following in vitro testing carried out by Fernandez et al., *L. delbrueckii* subsp. *lactis* UO 004 showed no significant degradation of mucin carbohydrate in samples incubated with fecal microbiota and without glucose [118]. The mucin degradation assay carried out on the plate presented the lack of mucinolytic zones around the tested LAB and the reference strain (*L. paracasei* Immunitas) [118]. Some LAB strains metabolize mucin glycoprotein components for multiplication. Wickström et al. demonstrated the ability of *L. fermentum* to degrade mucin MUC5B [119]. The obtained results suggested the potential use of mucins by the tested LAB strain as a nutrient source [119]. Pathogenic *S*. Typhimurium demonstrates the ability to degrade mucins and is a model organism used to study mucin degradation in various host tissues [120]. In our research, tested LAB strains showed a lack of ability to degrade mucins, suggesting the non-invasiveness of probiotic candidates needed for constructing a preparation to strengthen the immunity of honeybees.

None of the tested LAB strains displayed BSH activity. Opaque zones around the colonies were not observed. BSH activity in probiotic microorganisms is usually associated with the ability to lower serum cholesterol levels by participating in bile salt metabolism [121]. Bile salt hydrolysis is dependent on the host gut microbiota, however, excessive BSH activity can lead to pathogenic effects such as the increased risk of colon cancer and lipid malabsorption [122]. In in vitro assays conducted by Shehata et al., 8 out of 9 LAB strains tested demonstrated varying levels of BSH activity [123]. Four LAB isolates showed weak BSH activity and small zones of precipitation around the colonies [123]. Tanaka et al. evaluated the ability to hydrolyze bile salts by 300 LAB strains of the genus *Lactobacillus* and *Bifidobacterium* and the species *Streptococcus thermophilus*, *L. mesenteroides*, and *L. lactis* [122]. A lack of BSH activity was noted in *L. mesenteroides*, *S. thermophilus*, *L. lactis*, and some species of lactobacilli [122]. LAB strains isolated from environments where bile salts are not present, commonly lack BSH activity [124]. This observation coincides with the results of our study, where most of the tested strains were isolated from flowers and honey. The above-examined characteristics of probiotic candidates are important due to the likelihood of LAB entering the human body after consuming honeybee products produced by pollinators to which the probiotic was administered.

## 4. Conclusions

Honeybees are exposed to numerous factors affecting colony viability. Due to the environmental and economic importance of these insects, there is a growing need to strengthen their resistance using ecological methods. The above study allowed the selection of LAB strains with the most effective probiotic properties: *Pediococcus acidilactici* 21/1 (from freshly harvested fermented/spoiled honey), *P. pentosaceus* 5/2 (from *Weigela florida* DC. flowers) and 14/1 (from *Lavandula augustifolia* L. flowers), and *L. casei* 12AN (from human feces). LAB strains that demonstrated the most potent probiotic properties can be used to construct a protective ecological biopreparation for honeybees. Nevertheless, this research requires confirmation in vivo.

## Figures and Tables

**Table 1:** Antibiotic susceptibility profile of lactic acid bacteria (LAB) isolates.

Antibiotic (Concentration)	Antibiotic Susceptibility Profile of LAB *										
	*A. kunkeei* DSM 12361 12361	*P. acidilactici* 4/1	*P. pentosaceus* 5/2	*P. pentosaceus* 7/1	*P. acidilactici* 11/3	*P. pentosaceus* 14/1	*P. acidilactici* 18/1	*P. acidilactici* 21/1	*P. pentosaceus* 25/1	*P. pentosaceus* OK-S	*L. casei* 12AN
Ampicillin (10 µg)	S	S	MS	S	S	MS	S	S	S	S	S
Chloramphenicol (30 µg)	S	S	S	S	S	S	S	S	S	S	S
Chloramphenicol (50 µg)	S	S	S	S	S	S	S	S	S	S	S
Enrofloxacin (5 µg)	R	R	R	R	R	R	R	R	R	R	R
Erythromycin (30 µg)	S	S	S	S	S	S	S	S	S	S	S
Gentamicin (30 µg)	R	R	R	R	R	R	R	R	R	R	R
Kanamycin (30 µg)	R	R	R	R	R	R	R	R	R	R	R
Lincomycin (2 µg)	S	R	MS	MS	MS	MS	MS	MS	MS	MS	S
Lincomycin (15 µg)	S	R	S	S	S	MS	MS	S	S	MS	S
Oxytetracycline (30 µg)	MS	S	S	S	S	S	S	S	S	S	MS
Streptomycin (25 µg)	R	R	R	R	R	R	R	R	R	R	R
Sulfonamides (300 µg)	R	R	R	R	R	R	R	R	R	R	R
Tetracycline (30 µg)	MS	MS	MS	S	S	S	S	S	MS	S	MS
Tylosin (30 µg)	R	R	R	R	R	R	R	R	R	R	R
Vancomycin (30 µg)	R	R	R	R	R	R	R	R	R	R	R

* R: resistant, MS: moderately susceptible, S: susceptible.

## Data Availability

The data presented in this study are available in this article and from the corresponding authors upon reasonable request.

## Supplementary Materials

The following supporting information can be downloaded at: https://www.mdpi.com/article/10.3390/ani13061059/s1, Table S1: Survival of lactic acid bacteria (LAB) strains in the simulated digestive tract. Results are presented as mean cell viability ± standard deviation (SD), all values were divided by 10^6^ in order to simplify the table. LAB strains’ survival was compared to the *A. kunkeei* DSM 12361 using the Independent Samples *t* Tests. Significant differences in the LAB strains’ survival compared to the reference strain are indicated with colors and letters (only for the differences in the gastric juice); Table S2: Survival of lactic acid bacteria (LAB) strains in sugar syrups A, B, and C after 24 and 48 h of incubation. Results are presented as mean ± standard deviation (SD), and all values were divided by 10^7^ in order to simplify the table. Differences regarding the survival of LAB strains in sugar syrups were tested using the Kruskal–Wallis test (KWW test), followed by a multiple comparison test (MCT) to indicate significant differences between the groups at *p* < 0.05. Statistical differences in the survival of the LAB strains are indicated with letters: ^A, B^ indicating the groups with a statistical difference; Table S3: Impact of the cell-free culture supernatants (CFS) from lactic acid bacteria (LAB) strains on the viability of Caco-2 cells. Results are presented as mean (from 8 measurements) ± standard deviation (SD). The effect of CFS on Caco-2 cell survival rate has been tested using the Kruskal–Wallis test (KW test), followed by a multiple comparison test (MCT) to indicate significant differences between the groups at *p* < 0.05; significant differences are indicated with colors; Table S4: Impact of the cell-free culture supernatants (CFS) from lactic acid bacteria (LAB) strains on the viability of Caco-2 cells. Results are presented as mean (from 8 measurements) ± standard deviation (SD). The effect of CFS from LAB on Caco-2 cell survival rate has been tested using the Kruskal–Wallis test (KW test), followed by a multiple comparison test (MCT) to indicate significant differences between the groups at *p* < 0.05. Statistical differences in Caco-2 survival between LAB in each CFS concentration are indicated with ^A–N^; Table S5: Ability of *P. pentosaceus* 14/1 cell-free supernatant (CFS) to protect Caco-2 cells against the cytotoxicity of insecticides. Results for viability are presented as mean ± standard deviation (SD). The protective effect of CFS on Caco-2 cells against cytotoxicity has been tested using the Kruskal–Wallis test (KW test), followed by a multiple comparison test (MCT) to indicate significant differences between the groups at *p* < 0.05. Statistical differences are indicated with * (*p* = 0.003).

## Appendix A

List of culture vessels, chemicals, and other materials: 4-(2-hydroxyethyl)-1-piperazineethanesulphonic acid (HEPES), acetic acid, alpha-amylase, bile salts, chlorpyrifos (both of PESTANAL^®^ analytical standard purity, >99%), coumaphos, cysteine-hydrochloride, deMan–Rogosa–Sharp (MRS) broth and agar, dimethyl sulfoxide (DMSO), ethanol, fructose, glicodeoxycholic acid, glucose, HCl, high-glucose Dulbecco’s Modified Eagle Medium (DMEM), horseradish peroxidase, malachite green, mucin from the porcine stomach, NaOH, neutral red, pancreatin, Phosphate buffer saline (PBS), saccharose, sodium chloride (NaCl), pepsin, streptomycin–penicillin mixture for cell cultures, taurodeoxycholic acid, tetramethylbenzidine (TMB), trypan blue, Tryptic Soy Agar (TSA), Tryptic Soy Broth (TSB), and tylosin were purchased from Merck Life Science, Warsaw, Poland. Antibiotic discs (Oxoid) were purchased in Argenta, Poznań, Poland. AnaeroGen Atmosphere Generation Systems sachets, Foetal bovine serum (FBS), GlutaMAX^TM^, horse blood defibrinated, and TrypLE^TM^ Express were purchased from Thermo Fisher Scientific, Waltham, MA, USA. Cryobanks™ were from Copan Diagnostics Inc., Jefferson Avenue Murrieta, Murrieta, CA, USA. The Caco-2 cell line was from Cell Line Service GmbH, Eppelheim, Germany. Acetonitrile (HPLC grade) was from J.T. Baker (NJ, USA). In addition, 96-well plates, roux flasks, and serological pipettes (all from Greiner Bio-One GmbH Kremsmünster, Austria) were purchased in Biokom Systems, Janki, Poland. Syringe filters (0.22 µm pore size) were purchased from Labindex S.A., Warsaw, Poland.
