# Supplementary material for: Characterization and Protective Properties of Lactic Acid Bacteria Intended to Be Used in Probiotic Preparation for Honeybees (Apis mellifera L.)—An In Vitro Study
Source: Animals (Basel). 2023 Mar 15;13(6):1059. doi: 10.3390/ani13061059 (PMC10044574; doi:10.3390/ani13061059)
Supplement: Supplementary file 1 [file animals-13-01059-s001.zip › Table S1.pdf]

Characterization and Protective Properties of Lactic Acid Bacteria Intended to Be Used in Probiotic Preparation for Honeybees (*Apis mellifera* L.)—An In Vitro Study

Aleksandra Leska, Adriana Nowak, Justyna Rosicka-Kaczmarek, Małgorzata Ryngajłło, Karolina Henryka Czarnecka-Chrebelska

Table S1. Survival of lactic acid bacteria (LAB) strains in the simulated digestive tract. Results are presented as mean cell viability ± standard deviation (SD), all values were divided by 10^6 in order to simplify the table. LAB strains' survival was compared to the *A. kunkeei* DSM 12361 using the Independent Samples t Tests. Significant differences in the LAB strains' survival compared to the reference strain are indicated with colors and letters (only for the differences in the gastric juice).

LAB strain	Control 0h	Control 2h	Control 4h	Incubation in the gastric juice	Incubation in the intestinal juice
A. kunkeei DSM 12361	1422.50 ± 167.41	1335.00 ± 292.18	1605.00 ± 188.06	728.25 ± 99.85	1.10 ± 0.85
P. acidilactici 4/1	2775.00 ± 503.42	1832.50 ± 306.74	1932.50 ± 455.59	47.50 ± 4.95 [A]	0.30 ± 0.03
P. pentosaceus 5/2	1152.50 ± 371.07	1910.00 ± 461.23	1650.00 ± 84.85	39.00 ± 1.41 [B]	3.00 ± 0
P. acidilactici 18/1	535.00 ± 259.42	457.50 ± 251.45	432.50 ± 250.52	6.00 ± 1.41 [C]	0.89 ± 0.18
P. acidilactici 21/1	1060.00 ± 718.70	1247.50 ± 231.71	5537.50 ± 2384.68	763.00 ± 114.49	3.87 ± 1.63
P. pentosaceus 25/1	365.00 ± 173.11	290.00 ± 103.60	1447.50 ± 147.28	238.75 ± 134.81 [D]	1.07 ± 0.90
P. pentosaceus 7/1	1965.00 ± 351.99	2545.00 ± 197.40	2720.00 ± 129.61	875.00 ± 123.87	0.87 ± 0.84
P. acidilactici 11/3	1535.00 ± 283.84	4380.00 ± 1683.71	4257.50 ± 1349.48	1450.00 ± 578.79 [E]	0.01 ± 0.01
P. pentosaceus 14/1	2172.50 ± 409.66	2937.50 ± 260.82	2880.00 ± 165.93	705.25 ± 82.32	0.68 ± 0.38
P. pentosaceus OK-S	485.00 ± 193.30	407.50 ± 86.17	650.00 ± 47.61	374.75 ± 221.96 [F]	0.61 ± 0.46
L. casei 12AN	905.00 ± 188.06	2062.50 ± 455.95	2390.00 ± 143.99	530.25 ± 47.54 [G]	3.49 ± 3.69

Legend: statistically increased in comparison to the *A. kunkeei* DSM 12361 (yellow); statistically decreased in comparison to the *A. kunkeei* DSM 12361 (blue)

[A] p=0.0008, [B] p=0.0007, [C] p=0.0006, [D] p=0.001, [E] p=0.049, [F] p=0.027, [G] p=0.011, Independent Samples t Tests
