# Supplementary material for: Characterization and Protective Properties of Lactic Acid Bacteria Intended to Be Used in Probiotic Preparation for Honeybees (Apis mellifera L.)—An In Vitro Study
Source: Animals (Basel). 2023 Mar 15;13(6):1059. doi: 10.3390/ani13061059 (PMC10044574; doi:10.3390/ani13061059)
Supplement: Supplementary file 1 [file animals-13-01059-s001.zip › Table S2.pdf]

Characterization and Protective Properties of Lactic Acid Bacteria Intended to Be Used in Probiotic Preparation for Honeybees (*Apis mellifera* L.)—An In Vitro Study

Aleksandra Leska, Adriana Nowak, Justyna Rosicka-Kaczmarek, Małgorzata Ryngajłło, Karolina Henryka Czarnecka-Chrebelska

Table S2. Survival of lactic acid bacteria (LAB) strains in sugar syrups A, B, and C after 24 and 48 h of incubation. Results are presented as mean ± standard deviation (SD), and all values were divided by 10_7 in order to simplify the table. Differences regarding the survival of LAB strains in sugar syrups were tested using the Kruskal–Wallis test (KWW test), followed by a multiple comparison test (MCT) to indicate significant differences between the groups at $p < 0.05$. Statistical differences in the survival of the LAB strains are indicated with letters: $_{A, B}$ indicating the groups with a statistical difference.

**LAB strain**	**Control**			**Syrup A**		**Syrup B**		**Syrup C**		***p*-value KWW test**
	**0h**	**24h**	**48h**	**24h**	**48h**	**24h**	**48h**	**24h**	**48h**	
A. kunkeei DSM 12361	88.87 ± 48.09	305.25 ± 57.19	71.13 ± 25.07 ^{A}	58.53 ± 21.09	4.70 ± 1.78	34.50 ± 13.46	0.75 ± 0.30 ^{A}	62.25 ± 15.76	38.25 ± 4.86	p=0.002
P. acidilactici 18/1	55.37 ± 69.77	214.00 ± 82.93	103.43 ± 45.57 ^{A}	2.53 ± 1.67	0.16 ± 0.05 ^{A}	21.38 ± 12.88	0.24 ± 0.05	10.78 ± 6.42	0.22 ± 0.11	p=0.026
P. acidilactici 21/1	65.08 ± 77.89	207.48 ± 129.24	35.18 ± 8.00 ^{A,B}	30.45 ± 30.88	0.16 ± 0.05 ^{A}	53.40 ± 25.34	0.69 ± 0.01 ^{B}	2.29 ± 1.90	0.97 ± 0.31	p^{A}=0.008; p^{B}=0.023
P. pentosaceus 25/1	43.30 ± 30.66	133.50 ± 24.75	110.10 ± 25.49 ^{A}	4.70 ± 0.85	1.10 ± 0.27	18.20 ± 4.10	0.81 ± 0.25 ^{A}	28.00 ± 21.95	13.10 ± 8.01	p= 0.013
P. pentosaceus 5/2	132.12 ± 32.15	536.00 ± 177.04	213.75 ± 13.77 ^{A,B}	55.03 ± 5.59	6.75 ± 0.99 ^{A}	56.73 ± 3.93	6.75 ± 0.99 ^{B}	32.28 ± 4.53	12.05 ± 1.91	p^{A}=0.0178; p^{B}=0.0178
P. pentosaceus 7/1	194.16 ± 104.55	775.50 ± 108.33	474.75 ± 51.17 ^{A}	33.70 ± 5.53	8.15 ± 0.62	51.43 ± 6.89	8.15 ± 0.62	47.23 ± 6.77	4.25 ± 2.18 ^{A}	p=0.002
P. pentosaceus OK-S	76.32 ± 21.89	368.00 ± 39.19	3.60 ± 1.82	12.10 ± 9.42	0.98 ± 0.68	24.60 ± 19.87	1.23 ± 0.26	1.23 ± 0.26	1.43 ± 1.10	p>0.05
P. acidilactici 4/1	174.10 ± 315.59	696.25 ± 343.71	208.00 ± 100.66 ^{A}	7.23 ± 2.12	3.43 ± 2.81	23.00 ± 22.08	1.03 ± 0.05 ^{A}	38.08 ± 4.17	5.65 ± 1.91	p=0.008
P. acidilactici 11/3	176.06 ± 59.46	771.25 ± 182.53	533.00 ± 93.75 ^{A}	25.03 ± 12.00	24.83 ± 27.05	41.10 ± 7.79	1.03 ± 0.05 ^{A}	35.60 ± 9.97	11.00 ± 2.93	p=0.004
P. pentosaceus 14/1	176.23 ± 119.20	457.50 ± 71.36	438.25 ± 57.31 ^{A}	82.00 ± 13.98	27.80 ± 17.29	45.28 ± 29.79	3.40 ± 0.78 ^{A}	47.23 ± 8.81	11.15 ± 2.69	p=0.002
L. casei 12AN	209.05 ± 37.93	918.75 ± 94.11	464.25 ± 149.29 ^{A}	48.50 ± 33.97	53.13 ± 9.26	50.18 ± 2.21	4.78 ± 1.53 ^{A}	67.40 ± 14.15	22.95 ± 2.89	p=0.002
