# Supplementary material for: Characterization and Protective Properties of Lactic Acid Bacteria Intended to Be Used in Probiotic Preparation for Honeybees (Apis mellifera L.)—An In Vitro Study
Source: Animals (Basel). 2023 Mar 15;13(6):1059. doi: 10.3390/ani13061059 (PMC10044574; doi:10.3390/ani13061059)
Supplement: Supplementary file 1 [file animals-13-01059-s001.zip › Table S3.pdf]

Characterization and Protective Properties of Lactic Acid Bacteria Intended to Be Used in Probiotic Preparation for Honeybees (*Apis mellifera* L.)—An In Vitro Study

Aleksandra Leska, Adriana Nowak, Justyna Rosicka-Kaczmarek, Małgorzata Ryngajłło, Karolina Henryka Czarnecka-Chrebelska

Table S3. Impact of the cell-free culture supernatants (CFS) from lactic acid bacteria (LAB) strains on the viability of Caco-2 cells. Results are presented as mean (from 8 measurements) ± standard deviation (SD). The effect of CFS on Caco-2 cell survival rate has been tested using the Kruskal–Wallis test (KW test), followed by a multiple comparison test (MCT) to indicate significant differences between the groups at $p<0.05$; significant differences are indicated with colors.

LAB strain	Concentration of CFSs			
	**1%**	**5%**	**10%**	**20%**
A. kunkeei DSM 12361	115.73 ± 7.75	126.25 ± 13.72	114.53 ± 11.86	78.72 ± 6.66
P. acidilactici 4/1	93.28 ± 7.2	97.91 ± 10.03	94.39 ± 7.12	34.91 ± 10.46
P. pentosaceus 5/2	103.44 ± 9.44	109.3 ± 9.72	124.42 ± 6.71	89.81 ± 8.74
P. pentosaceus 7/1	101.65 ± 6.89	116.65 ± 4.69	100.24 ± 8.7	45.80 ± 8.83
P. acidilactici 11/3	105.68 ± 11.55	108.71 ± 6.88	91.12 ± 15.5	29.80 ± 8.12
P. pentosaceus 14/1	97.5 ± 7.41	112.16 ± 12.23	110.53 ± 10.05	36.58 ± 5.89
P. acidilactici 18/1	101.08 ± 8.81	108 ± 7.86	94.49 ± 17.49	30.26 ± 3.51
P. acidilactici 21/1	102.28 ± 5.66	109.54 ± 6.59	100.06 ± 9.62	34.09 ± 3.08
P. pentosaceus 25/1	102.73 ± 7.82	101.47 ± 10.09	107.6 ± 7.38	75.65 ± 15.12
L. casei 12AN	164.53 ± 17.88	144.41 ± 10.29	157.26 ± 24.8	30.17 ± 3.48
P. pentosaceus OK-S	97.48 ± 6.31	95.7 ± 7.55	90.53 ± 20.46	30.92 ± 5.05

Legend: significantly increased Caco-2 cells survival ($p<0.05$. KW test) | significantly decreased Caco-2 cells survival ($p<0.05$. KW test)
