# Supplementary material for: Characterization and Protective Properties of Lactic Acid Bacteria Intended to Be Used in Probiotic Preparation for Honeybees (Apis mellifera L.)—An In Vitro Study
Source: Animals (Basel). 2023 Mar 15;13(6):1059. doi: 10.3390/ani13061059 (PMC10044574; doi:10.3390/ani13061059)
Supplement: Supplementary file 1 [file animals-13-01059-s001.zip › Table S4.pdf]

Characterization and Protective Properties of Lactic Acid Bacteria Intended to Be Used in Probiotic Preparation for Honeybees (*Apis mellifera* L.)—An In Vitro Study

Aleksandra Leska, Adriana Nowak, Justyna Rosicka-Kaczmarek, Małgorzata Ryngajłło, Karolina Henryka Czarnecka-Chrebelska

Table S4. Impact of the cell-free culture supernatants (CFS) from lactic acid bacteria (LAB) strains on the viability of Caco-2 cells. Results are presented as mean (from 8 measurements) ± standard deviation (SD). The effect of CFS from LAB on Caco-2 cell survival rate has been tested using the Kruskal–Wallis test (KW test), followed by a multiple comparison test (MCT) to indicate significant differences between the groups at $p<0.05$. Statistical differences in Caco-2 survival between LAB in each CFS concentration are indicated with $^{A\text{-}N}$.

**LAB strain**	**Concentration of CFSs**			
	**1%**	**5%**	**10%**	**20%**
A. kunkeei DSM 12361	115.73 ± 7.75 ^{A,B}	126.25 ± 13.72 ^{A,B}	114.53 ± 11.86	78.72 ± 6.66 ^{A,B,C,D}
P. acidilactici 4/1	93.28 ± 7.2 ^{A,C}	97.91 ± 10.03 ^{A,C}	94.39 ± 7.12 ^{A,B}	34.91 ± 10.46 ^{E}
P. pentosaceus 5/2	103.44 ± 9.44	109.30 ± 9.72	124.42 ± 6.71 ^{A,C,D,E}	89.81 ± 8.74 ^{E,F,G,H,I,J,K}
P. pentosaceus 7/1	101.65 ± 6.89 ^{D}	116.65 ± 4.69 ^{D}	100.24 ± 8.70 ^{F}	45.80 ± 8.83
P. acidilactici 11/3	105.68 ± 11.55	108.71 ± 6.88	91.12 ± 15.50 ^{C,G}	29.80 ± 8.12 ^{A,F,K}
P. pentosaceus 14/1	97.50 ± 7.41 ^{E}	112.16 ± 12.23	110.53 ± 10.05	36.58 ± 5.89
P. acidilactici 18/1	101.08 ± 8.81 ^{F}	108.00 ± 7.86 ^{E}	94.49 ± 17.49 ^{D,H}	30.26 ± 3.51 ^{B,G,L}
P. acidilactici 21/1	102.28 ± 5.66	109.54 ± 6.59	100.06 ± 9.62 ^{I}	34.09 ± 3.08 ^{H}
P. pentosaceus 25/1	102.73 ± 7.82	101.47 ± 10.09 ^{F}	107.60 ± 7.38	75.65 ± 15.12 ^{K,L,M,N}
L. casei 12AN	164.53 ± 17.88 ^{C,D,E,F,G}	144.41 ± 10.29 ^{C,E,F,G}	157.26 ± 24.80 ^{B,F,G,H,I,J}	30.17 ± 3.48 ^{C,I,M}
P. pentosaceus OK-S	97.48 ± 6.31 ^{B,G}	95.7 ± 7.55 ^{B,D,G}	90.53 ± 20.46 ^{E,J}	30.92 ± 5.05 ^{D,J,N}
p-values (KW test)	^{A} 0.0029; ^{B} 0.05;<br>^{C} 0.000; ^{D} 0.0351;<br>^{E} 0.0008; ^{F} 0.0077;<br>^{G} 0.0007	^{A} 0.0097; ^{B} 0.0026;<br>^{C} 0,000; ^{D} 0.0272;<br>^{E} 0.0426; ^{F} 0.0008;<br>^{G} 0.000	^{A} 0.0048; ^{B} 0.0002;<br>^{C} 0.0073; ^{D} 0.0351;<br>^{E} 0.0065; ^{F} 0.0137;<br>^{G} 0.0003; ^{H} 0.0021;<br>^{I} 0.0127; ^{J} 0.0003	^{A} 0.002; ^{B} 0.0066;<br>^{C} 0.0052; ^{D} 0.0062;<br>^{E} 0.0069; ^{F} 0.0001;<br>^{G} 0.0005; ^{H} 0.0303;<br>^{I} 0.0004; ^{J} 0.0005;<br>^{K} 0.0023; ^{L} 0.0074;<br>^{M} 0.0059; ^{N} 0.007
