# Supplementary material for: Characterization and Protective Properties of Lactic Acid Bacteria Intended to Be Used in Probiotic Preparation for Honeybees (Apis mellifera L.)—An In Vitro Study
Source: Animals (Basel). 2023 Mar 15;13(6):1059. doi: 10.3390/ani13061059 (PMC10044574; doi:10.3390/ani13061059)
Supplement: Supplementary file 1 [file animals-13-01059-s001.zip › Table S5.pdf]

Characterization and Protective Properties of Lactic Acid Bacteria Intended to Be Used in Probiotic Preparation for Honeybees (*Apis mellifera* L.)—An In Vitro Study

Aleksandra Leska, Adriana Nowak, Justyna Rosicka-Kaczmarek, Małgorzata Ryngajłło, Karolina Henryka Czarnecka-Chrebelska

Table 5 Ability of *P. pentosaceus* 14/1 cell-free supernatant (CFS) to protect Caco-2 cells against the cytotoxicity of insecticides. Results for viability are presented as mean ± standard deviation (SD). The protective effect of CFS on Caco-2 cells against cytotoxicity has been tested using the Kruskal–Wallis test (KW test), followed by a multiple comparison test (MCT) to indicate significant differences between the groups at $p<0.05$. Statistical differences are indicated with * ($p=0.003$).

	**Chlorpyrifos 50 μg/mL**	**Chlorpyrifos 25 μg/mL**	**Coumaphos 25 μg/mL**	**Coumaphos 12.5 μg/mL**
Without initial incubation with CFS	65.83 ± 9.47	61.63 ± 12.28	45.95 ± 5.35*	60.5 ± 4.81
Incubation with 0.1 mg/mL CFS	61.21 ± 19.12	60.45 ± 13.84	58.09 ± 3.72	63.45 ± 7.82
Incubation with 1.0 mg/mL CFS	68.61 ± 4.63	64.00 ± 3.93	63.18 ± 2.84	79.82 ± 16.91*
